# Pattern and correlates of coronary artery disease among patients referred for coronary angiography at Abuad Multisystem Hospital, Ado-Ekiti, Nigeria: a descriptive cross-sectional study

**DOI:** 10.11604/pamj.2025.50.55.45726

**Published:** 2025-05-18

**Authors:** Oladipo Ayoola Olanipekun, Stephen Olawale Oguntola, Adekunle Kayode Agoke, Adedayo Hakeem Oyebanji, Olamide Nelson Ogidan, Akinola Akinmade, Dhirendrakumar Jayantilal Dave, Anthony Olubunmi Akintomide, Michael Sanusi, Kamar Tayo Adeleke

**Affiliations:** 1 Department of Medicine, Cardiology Unit, Faculty of Clinical Sciences, College of Medicine and Health Sciences, Afe Babalola University, Ado-Ekiti, Ekiti State, Nigeria,; 2 Department of Medicine, Nephrology Unit, Faculty of Clinical Sciences, College of Medicine and Health Sciences, Afe Babalola University, Ado-Ekiti, Ekiti State, Nigeria,; 3 Department of Internal Medicine, Cardiology Unit, Federal Medical Centre, Owo, Ondo State, Nigeria,; 4 Department of Paediatrics, Paediatric Cardiology Unit, Faculty of Clinical Sciences, College of Medicine and Health Sciences, Afe Babalola University, Ado-Ekiti, Ekiti State, Nigeria,; 5 Department of Surgery, Orthopaedics Surgery Unit, Faculty of Clinical Sciences, College of Medicine and Health Sciences, Afe Babalola University, Ado-Ekiti, Ekiti State, Nigeria,; 6 Department of Surgery, Cardiovascular Surgery Unit, Faculty of Clinical Sciences, College of Medicine and Health Sciences, Afe Babalola University, Ado-Ekiti, Ekiti State, Nigeria,; 7 Department of Medicine, Cardiology Unit, Faculty of Clinical Sciences, College of Health Sciences, Obafemi Awolowo University, Osun State, Nigeria,; 8 Abuad Tristate Heart and Vascular Centre, Ado-Ekiti, Ekiti State, Nigeria,; 9 Tristate Healthcare System, Eti-Osa, Lagos, Nigeria

**Keywords:** Coronary artery disease, coronary angiography, diabetes mellitus, erythrocyte sedimentation rate

## Abstract

**Introduction:**

coronary artery disease (CAD) is a common cardiovascular disease (CVD) and a notable cause of mortality worldwide. Angiographic diagnosis of CAD is not widely available in sub-Saharan Africa. We assessed the pattern and correlates of CAD among patients referred for coronary angiography in Ado-Ekiti, Nigeria.

**Methods:**

patients with clinical suspicion of CAD who were referred for coronary angiography at Afe Babalola University Multi-System Hospital (AMSH) from August 2019 to December 2020 were recruited into the study after a written informed consent was obtained. Serum fasting lipids, electrolytes, urea and creatinine and erythrocyte sedimentation rate (ESR) were assessed. All patients had coronary angiography.

**Results:**

forty-six (46) patients were recruited into the study. The mean age among patients who had CAD was 52.12 years (±10.34). There were 15 (32.6%) females in the study population. CAD was confirmed in 18/46 (39.1%) patients. Triple vessel disease (TVD) was seen in 9/18 (50.0%) of patients who had CAD, hence, the most prevalent CAD disease pattern. ESR was significantly prolonged among patients who had CAD compared to patients who did not have CAD on univariate analysis while diabetes mellitus was an independent risk factor for CAD and conferred a greater than 7-fold risk for CAD (aOR: 7.28, 95% CI 1.15-46.16; p = 0.035).

**Conclusion:**

diabetes mellitus was an independent risk factor for CAD. Triple vessel disease was the most common pattern of CAD in our study. ESR was associated with CAD.

## Introduction

Globally, cardiovascular disease (CVD) is the most common cause of mortality [1]. There has been a rapid increase in the number of deaths attributable to CVD from 12.5 million in 1990 to 17.9 million in 2015 [2]. In 2016, CVD was responsible for 17.95 million deaths, which accounted for 31% of all global deaths; 85% of all cardiovascular deaths were due to coronary artery disease (CAD) and stroke [1]. Coronary artery disease is the most common type of CVD and possibly the most lethal, responsible for the death of more than 365,000 people in the United States in 2019 [3]. Previously, CAD was thought to be a problem in developed countries, however, the current trend has shown a rising prevalence in Nigeria and other West African countries [4-7]. A pioneer cross-sectional study that was done in Lagos, Nigeria, confirmed CAD in 52.6% (80/152) of patients referred for diagnostic coronary angiography for suspected coronary artery disease [7]. The prevalence of CAD reported in the study was 2.8% of all medical cases seen during the study duration. The study reported a high prevalence of CAD risk factors that were similar to those documented in developed countries [8,9]. The similarity in the CAD risk factors reported in developed and developing countries may be because of increasing westernization in sub-Saharan countries.

Despite the increasing trend of CAD in sub-Saharan Africa, there remains a major challenge to the paucity of diagnostic and interventional facilities in West African countries, including Nigeria [7]. Currently in Nigeria, only a few health institutions are adequately equipped for diagnostic and interventional coronary intervention. Most of these procedures are being done in private hospitals because most government hospitals lack such facilities, probably because of sub-optimal funding. Earlier cross-sectional studies in Nigeria on coronary angiographic findings and profiles of patients reported the frequencies of risk factors among patients with CAD, but not the predictors of CAD among patients being evaluated for coronary heart disease [7,8]. This study aimed to assess the pattern and correlates of CAD among patients who were referred for coronary angiography at Abuad Multisystem Hospital.

## Methods

**Study design and setting:** this was a retrospective and prospective (cross-sectional) study that included all the patients with clinical suspicion of CAD who had coronary angiography at Abuad Multi-System Hospital (AMSH) Ado-Ekiti, Ekiti State, Nigeria. The study location was AMSH Ado-Ekiti, Ekiti State, Nigeria. AMSH is a 400-bed tertiary hospital. AMSH receives referrals from general and tertiary hospitals in Southwestern Nigeria. The Cardiovascular Unit is equipped with modern facilities for invasive and non-invasive cardiac evaluation. The Cardiac Unit has a cardiac catheterization laboratory where all the coronary procedures are carried out. The cardiac unit also has a modular fully automated cardiac theatre and a 4-bed cardiac ICU. The study duration was from August 2019 to December 2020.

**Study population:** all the patients with clinical suspicion of CAD who had coronary angiography at ABUAD Multi-System Hospital (AMSH) Ado-Ekiti, Ekiti State, Nigeria.

**Data collection:** the data was retrieved from the AMSH electronic medical records and this included sociodemographic and clinical characteristics of the study population, baseline test results such as electrolytes, urea and creatinine, lipid profile, erythrocyte sedimentation rate (ESR) and presence and severity of CAD from the coronary angiography report.

**Sample size:** the data of all the 46 patients who had coronary angiography at AMSH, Ado-Ekiti, Nigeria, within the study period was analysed.

**Definition of variables:** CAD: coronary artery disease; evidence of SVD (single vessel disease - presence of plaques causing narrowing of one major coronary artery), DVD (double vessel disease - presence of plaques causing narrowing of two major coronary arteries), TVD (triple vessel disease - presence of plaques causing narrowing of three major coronary arteries) or MDD (mild diffuse disease - presence of plaques in a large section of coronary artery that is not significant enough to have any haemodynamic effect on coronary blood flow) in the coronary artery. DM (diabetes mellitus): patients on treatment for DM. Hypertension: patients on treatment for hypertension.

Serum lipid profile (total cholesterol-TC, low-density lipoprotein-LDL-C, triglyceride-TG, high-density lipoprotein-HDL-C), electrolytes, urea and creatinine were analyzed using ABBOT C4000 ARCHITECT PLUS (Toshiba Medical Systems Corporation, Japan). The erythrocyte sedimentation rate was determined by the Westergreen method. Coronary angiography was performed using TOSHIBA INFINIX 8000, CAS-880A, and BLA-900A (Toshiba Medical Systems Corporation, Japan). The left and right main coronary arteries were cannulated with appropriate-size catheters using the modified Judkins technique. Percentage stenosis was determined using a pre-installed electronic calliper system. Estimated GFR was calculated using the 2009 CKD-EPI formula [10].

**Statistical analysis:** Stata version 13.1, (StataCorp, USA) was used for statistical analysis. Categorical variables were expressed as frequencies and percentages and compared using the chi-square test. Mean, median, histogram and test of normality including Shapiro-Wilk and S-K tests were performed on all continuous variables and normally distributed data were presented as mean ± SD while non-normally distributed data were presented as median and interquartile range (IQR). Comparisons were performed between participants who had CAD and those who did not, using the student t-test for normally distributed data and the Wilcoxon rank-sum test for non-normally distributed data. A univariable logistic regression analysis was performed, followed by a multivariable binary logistic regression analysis to determine the predictors of CAD. The multivariable model was built in a stepwise backward elimination technique using explanatory variables with univariable p-value < 0.2. Some variables, such as age, male gender, diabetes mellitus and hypertension were chosen *a priori* based on the literature search and experience of the researchers. Multicollinearity was checked using the variance inflation factor (VIF). The goodness of fit of the multivariable model was determined by the Hosmer-Lemeshow test. Significance was taken as p < 0.05.

**Ethical considerations:** the study was approved by the Ethics Review Committee of AMSH, with protocol number AMSH/REC/SOO/120.

## Results

A total of 46 patients had coronary angiography in AMSH during the review period and all were used in the analysis. There were 31 (67.4) males and 15 (32.6%) females in the study population. The mean age was 56.88 years (±13.08) among patients who had no CAD and 52.12 years (±10.34) among patients who had CAD (Table 1). Precordial chest pain was the presenting complaint in 25/46 (54.4%) of the study population while alcohol use was seen in 17/46 (40.0%). Only one patient was a smoker. Hypertension was seen in 39/46 (84.8%); among the hypertensives, 17/39 (43.6%) had CAD. There were ten patients who had diabetes mellitus in the study population. Significantly increased frequency of CAD was seen in patients who had diabetes mellitus (7/10, 70.0%) compared to patients who did not have diabetes ((7/10, 70%) vs (11/36, 30.6%); p = 0.033). Significantly prolonged ESR was seen among patients who had CAD compared to patients who did not have CAD. No significant differences were seen when laboratory markers such as haematocrit, platelet count, serum sodium and potassium levels, bicarbonate, serum lipoprotein levels and urea and creatinine levels were compared between patients who had CAD and patients who did not have CAD.

**Table 1:** sociodemographic and clinical characteristics of the study population

Parameter	CAD absent (%)	CAD present (%)	p-value
Age	56.88 ± 13.08	52.12 ± 10.34	0.175*
**Gender**			
female	7 (25)	8 (44.4)	0.170^β^
male	21 (75)	10 (55.6)	
**Chest pain**			
Absent	15 (53.6)	6 (33.3)	0.179^β^
Present	13 (46.4)	12 (66.7)	
**Alcohol use**			
No	19 (67.9)	10 (55.6)	0.399^β^
Yes	9 (32.1)	8 (44.4)	
**Cigarette smoking**			
No	27 (96.4)	18 (100.0)	1.000^ɸ^
Yes	1 (3.6)	0 (0.0)	
**Hypertension**			
Absent	6 (21.4)	1 (5.6)	0.199^ɸ^
Present	22 (78.6)	17 (94.4)	
**Diabetes**			
Absent	25 (89.3)	11 (61.1)	0.033^ɸ^
Present	3 (10.7)	7 (41.2)	
CAD	28 (60.9)	18 (39.1)	
Coronary revascularization		6 (33.3)	
Stent		3 (50.0)	
CABG		3 (50.0)	
**Coronary arterial dominance**			
RCA	24.0 (85.7)	12.0 (66.7)	0.204^ɸ^
LCX	3.0 (10.7)	3.0 (16.7)	
Co-dominance	1.0 (3.6)	3.0 (16.7)	
Haematocrit (%)	40.07 ± 5.98	39.30 ± 6.04	0.672*
Platelet	218.71 ± 54.12	257.78 ± 81.18	0.056*
Sodium (mmol/l)	136.93 ± 3.43	137.00 ± 5.45	0.957*
Potassium (mmol/l)	3.64 ± 0.45	3.81 ± 0.48	0.235*
Bicarbonate (mmol/l)	22.46 ± 3.25	22.78 ± 3.19	0.749*
TC (mmol/l)	4.71 ± 0.99	4.23 ± 1.43	0.376*
LDL-C (mmol/l)	3.07 ± 0.924	2.56 ± 1.27	0.301*
HDL-C (mmol/l)	1.09 ± 0.27	1.21 ± 0.38	0.422*
TG (mmol/l)	1.26 ± 0.47	1.03 ± 0.47	0.289*
ESR (mm/hr)	10.0 (5.0 - 18.0)	26.0 (19.0 - 50.0)	0.043^#^
Urea (mmol/l)	4.5 (3.5 - 6.3)	4.7 (3.3 - 5.9)	0.761^#^
Creatinine (µmol/l)	101.3 (77.3 - 116.3)	91.6 (66.9 - 105.0)	0.184^#^
GFR (ml/min/1.73m^2^)	81.0 (64.0 - 96.5)	88.0 (66.0 - 104.0)	0.437^#^

Mean ± standard deviation, t-test; ^#^: median (interquartile range), Wilcoxon rank-sum test; ^ɸ^: frequency (percentage), fisher’s exact; ^β^: frequency (percentage), Pearson chi-square test; CAD: coronary artery disease; CABG: coronary artery bypass surgery; RCA: right coronary artery; LCX: left circumflex artery; TC: total cholesterol; LDL-C: low density lipoprotein cholesterol; HDL-C: high density lipoprotein cholesterol; TG: triglyceride; ESR: erythrocyte sedimentation rate; GFR: glomerular filtration rate

In our study, the prevalence of angiographically diagnosed CAD was 39.1% (n = 18/46) among patients referred for coronary angiography; only 6 of these patients had coronary revascularization therapies including coronary artery stenting (3/6, 50.0%) and Coronary Artery Bypass Grafting (CABG) (3/6, 50.0%). Patients with non-obstructive disease were offered medical management. The right coronary artery (RCA) was the dominant coronary artery in 78% (n = 36/46) of the study population while codominance was seen in 2.2% (n = 1/46). Triple vessel disease (TVD) was seen in 9/18 (50.0%) of patients who had CAD, hence, the most prevalent CAD disease pattern was based on the number of vessels affected (Table 2). Single vessel disease (SVD) was seen in 33.3% (6/18) of patients who had CAD.

**Table 2:** coronary artery disease by number of vessels

Number of vessels	Frequency	Percentage (%) of study population (n = 46)	Percentage (%) of patients that had CAD (n = 18)
No CAD	28	60.9	
SVD	6	13.0	33.3
DVD	3	6.5	16.7
TVD	9	19.6	50.0

CAD: coronary artery disease; SVD: single vessel disease; DVD: double vessel disease; TVD: triple vessel disease

Coronary arterial luminal stenosis of ≥ 90% was the most common in all the arterial territories (Table 3). The prevalence of isolated proximal lesions was 13.0% (6/46), 4.4 (2/46) and 6.5% (3/46) in left anterior descending (LAD), left circumflex (LCX) and RCA respectively. Combined coronary artery lesions were found in varying combinations in all arterial territories.

**Table 3:** pattern and severity of coronary artery disease in the study population

Parameters	LAD	LCX	RCA
**Degree of stenosis (%)**			
No lesion	28 (60.9)	40 (87.0)	37 (80.4)
<50%	3 (6.5)	1 (2.2)	2 (4.4)
50-69%	1 (2.2)		
70-89	1 (2.2)	1 (2.2)	1 (2.2)
≥90	13 (28.3)	4 (8.7)	6 (13.0)
**Distribution of lesion within vessel**			
Normal	28 (60.9)	40 (87.0)	37 (80.4)
Proximal	6 (13.0)	2 (4.4)	3 (6.5)
Mid	4 (8.7)	2 (4.4)	3 (6.5)
Distal	1 (2.2)		1 (2.2)
**Diffuse**			
Ostia	1 (2.2)		
Mid + distal	1 (2.2)		
Proximal + distal		2 (4.4)	1 (2.2)
Proximal + mid + distal	1 (2.2)		
Ostia + mid	1 (2.2)		
Ostia + proximal	1 (2.2)		
MDD	2 (4.4)		1 (2.2)

LAD: left anterior descending; LCX: left circumflex artery; RCA: right coronary artery; MDD: mild diffuse disease

The multivariable logistic regression showed that diabetes mellitus was an independent risk factor of CAD (Table 4). There was a 7.28-fold risk of CAD among patients with diabetes mellitus.

**Table 4:** multivariable logistic regression for risk factors for coronary artery disease

Risk factors	Unadjusted OR (95% CI)	p	Adjusted OR (95% CI)	p
Age^#^	1.03 (0.98 - 1.08)	0.284	1.03 (0.96 - 1.10)	0.409
Male gender	0.42 (0.12 - 1.47)	0.174	0.50 (0.11 - 2.37)	0.382
Chest pain	2.31 (0.67 - 7.92)	0.183	5.34 (0.91 - 31.41)	0.064
DM	5.30 (1.15 - 24.42)	0.032*	7.28 (1.15 - 46.16)	0.035*
Hypertension	4.64 (0.02 - 1.38)	0.174	9.55 (0.56 - 164.11)	0.120

# :linear trend; *:statistically significant, p < 0.05; OR: odd ratio; DM: diabetes mellitus

## Discussion

Access to coronary angiography still remains limited in sub-Saharan Africa [7]. This is due to the limited number of cardiac catheterization laboratories, especially in the West African sub-continent. Coronary artery disease was previously thought to be uncommon in sub-Saharan African countries, however, findings from pioneer studies in Nigeria which showed a rising prevalence of CAD have invalidated the earlier reports [7,8]. These earlier studies did not report the predictors of CAD among Nigerians. We evaluated the pattern and correlates of angiographic findings among patients with clinical suspicion of CAD who were referred for coronary angiography at Abuad Multisystem Hospital.

In our study, CAD was confirmed in 39.1% (18/46) of patients referred for coronary angiography during the study period. Contrary to the previous belief that CAD is uncommon in sub-Saharan Africa, our finding supports the current trends of reports that showed that the prevalence of ischemic heart disease (IHD) is rising among Black Africans [7,8]. A similar study by Johnson *et al*. reported 52.6% confirmed CAD cases among patients with suspected coronary heart disease who were referred for coronary angiography in a private cardiac catheterization laboratory in Nigeria [7].

Our study showed that LAD is the most frequently affected vessel and the most common site for multi-segmental obstruction. The frequency of distribution of lesions in the epicardial vessels followed a pattern similar to what was published in previous studies [11,12]. This finding suggests that additional haemodynamic factors, such as hypertension may play a role in the pathogenesis of CAD. In our study, 43.6% of hypertensives had CAD. Another significant finding that is common to all the major epicardial vessels is that most lesions were located within the proximal segment of the vessel. The arteriosclerotic lesion has been shown to develop commonly close to the branch point of major epicardial vessels [13]. This may be attributable to high flow dynamics within the proximal segment, which is close to the branch point of the vessel making this segment more predisposed to endothelial injury and dysfunction, one of the primary events in arteriosclerosis [13].

We demonstrated severe stenosis of >90% in most of the epicardial vessels and the most common site was the LAD. The high frequency of severe disease in patients with CAD is possibly due to the late presentation of our patients for diagnostic catheterization. Two main factors may preclude early presentation. Firstly, cardiac catheterization service is currently available in a few tertiary centres in Nigeria. Secondly, there is a lack of financial capacity to access such facilities in centres where they exist.

In our study, we found that triple vessel disease was the most common CAD pattern seen in half of the patients with CAD. This is similar to previous reports of angiographic findings among African Americans and Nigerians [8,14]. This finding can be explained by the clinical profile of the study participants because nearly a quarter of the participants were diabetic, 70% of whom had CAD. A study compared the severity of CAD between type 2 diabetic patients and non-diabetic patients and found that patients with diabetes had more severe atherosclerotic lesions in their coronary arteries [15]. A more recent study assessed the severity of CAD among non-diabetics, pre-diabetics and patients with type 2 diabetes. The study found that pre-diabetes and patients with diabetes were more likely to have extensive CAD; pre-diabetes was also reported as an independent risk factor for CAD [16]. A systematic review that assessed the effect of diabetes on the progression of coronary atherosclerosis reported that diabetes mellitus was associated with severe atherosclerosis and accelerated plaque progression despite medical therapies [17]. Diabetes mellitus has been associated with other cardiovascular risk factors such as hypertension, obesity and dyslipidaemia and with increased CVD [18]. It is, therefore, conceivable that diabetes mellitus was independently associated with CAD in our study. The relationship between diabetes and extensive CAD has also been explained by the increased risk of endothelial dysfunction among patients with diabetes [19]. It is, therefore, imperative to ensure early diagnosis of DM and maintenance of adequate blood glucose control to reduce the risk of CAD.

Erythrocyte sedimentation rate (ESR) has been used as a marker of chronic inflammation, which is one of the main pathogenic mechanisms of coronary atherosclerosis [20]. We found a significantly prolonged ESR among patients who had CAD compared to patients who did not have CAD. Several studies have documented prolonged ESR in patients with CAD compared to patients who do not have CAD [20-22]. In resource-poor settings where there is no rapid access to diagnostic cardiac markers, prolonged ESR in the presence of typical clinical features may increase the clinical suspicion of CAD, which may help to triage patients into high- and low-probability for CAD.

Limitations of the study include the small sample size and the retrospective/cross-sectional nature of the study design, which reduced the power of the study and limited the number of variables available for statistical analysis respectively.

## Conclusion

Diabetes mellitus was an independent predictor of CAD. Triple vessel disease was the most common pattern among our patients who had CAD. Erythrocyte sedimentation rate was associated with CAD.

### What is known about this topic

- Coronary artery disease is common in developed countries;
- Diabetes is one of the risk factors for CAD.

### What this study adds

- Our study found that DM was an independent risk factor for CAD after adjusting for age, male gender, chest pain and hypertension;
- Triple vessel disease was the most common pattern among our patients who had CAD;
- Erythrocyte sedimentation rate levels were associated with CAD.
